# A case study of an adolescent according to A Model of Children’s Adjustment to Parental Cancer

**DOI:** 10.1590/1980-220X-REEUSP-2022-0188en

**Published:** 2022-09-12

**Authors:** Ana Filipa Domingues Sousa, Diana Gabriela Simões Marques Santos, Cristina Raquel Baptista Costeira, Maria Margarida da Silva Reis dos Santos Ferreira, Maria de Lurdes Lopes de Freitas Lomba

**Affiliations:** 1 Universidade do Porto, Instituto de Ciências Biomédicas Abel Salazar, Porto, Portugal.; 2 Instituto Português de Oncologia de Coimbra, Coimbra, Portugal.; 3 Unidade de Investigação em Ciências da Saúde: Enfermagem, Escola Superior de Enfermagem de Coimbra, Coimbra, Portugal.; 4 Centro Hospitalar e Universitário de Coimbra, Coimbra, Portugal.; 5 Instituto Politécnico de Leiria, Escola Superior de Saúde, Leiria, Portugal.; 6 Center for Innovative Care and Health Technology, Leiria, Portugal.; 7 Centro de Investigação em Tecnologias e Serviços de Saúde, Porto, Portugal.; 8 Escola Superior de Enfermagem do Porto, Porto, Portugal.

**Keywords:** Adolescent, Neoplasms, Nursing, Models, Theoretical, Adolescente, Neoplasias, Enfermería, Modelos Teóricos, Adolescente, Neoplasias, Enfermagem, Modelos Teóricos

## Abstract

**Objective:**

To describe and analyze the experience of an adolescent experiencing parental cancer, based on A Model of Children’s Adjustment to Parental Cancer, and to prescribe nursing interventions in classified language.

**Method:**

This is a single case study, qualitative, of a 16-year-old adolescent experiencing maternal cancer. We analyzed a semi-structured interview, based on a script conceptualized by the selected theoretical model. Data processing took place through content analysis. Authorization was obtained from the Research Ethics Committee TI 25/2020.

**Results:**

The analysis of the adolescent’s interview allowed identifying categories in agreement with the model variables. Psychosocial adjustment dimensions and stress response symptoms, such as academic performance and somatic symptoms, were recognized in the adolescent’s adjustment process. Nursing interventions will focus on education and support.

**Conclusion:**

The theoretical model contributed to assess the needs of adolescents experiencing parental cancer, allowing nursing interventions to be prescribed in classified language that consider moderating and mediating variables, promoting adjustment. The model proved to be suitable for future interventions for adolescents experiencing similar situations.

## RESUMO

**Objetivo:**

descrever e analisar experiência de um adolescente a vivenciar cancro parental, fundamentado no Modelo Teórico do Ajuste dos Adolescentes ao Cancro Parental, e prescrever intervenções de enfermagem em linguagem classificada.

**Método:**

estudo de caso único, tipo qualitativo, de adolescente de 16 anos de idade a vivenciar cancro da mãe. Analisou-se uma entrevista semiestruturada, baseada em um roteiro concetualizado pelo modelo teórico selecionado. Tratamento de dados ocorreu através de análise de conteúdo. Obteve-se autorização da Comissão de Ética TI 25/2020.

**Resultados:**

a análise da entrevista do adolescente permitiu identificar categorias concordantes com as variáveis do modelo. As dimensões de ajuste psicossocial e sintomas de resposta ao estress, como performance acadêmica e sintomas somáticos, foram reconhecidas no processo de ajuste do adolescente. As intervenções de enfermagem serão focadas na educação e apoio.

**Conclusão:**

o modelo teórico contribuiu para avaliar as necessidades do adolescente a vivenciar cancro parental, permitindo prescrever intervenções de enfermagem em linguagem classificada que considerem variáveis moderadoras e mediadoras, promovendo-se o ajuste. O modelo demonstrou ser adequado para futuras intervenções a adolescentes a experienciar situações semelhantes.

## RESUMEN

**Objetivo:**

Describir y analizar la experiencia de un adolescente con cáncer en los padres, a partir del Modelo Teórico de Adaptación del Adolescente al Cáncer en los Padres, y prescribir intervenciones de enfermería en lenguaje clasificado.

**Método:**

Estudio de caso único, cualitativo, de una adolescente de 16 años con cáncer materno. Se analizó una entrevista semiestructurada, a partir de un guión conceptualizado por el modelo teórico seleccionado. El procesamiento de datos se llevó a cabo a través del análisis de contenido. Se obtuvo autorización del Comité de Ética TI 25/2020.

**Resultados:**

El análisis de la entrevista adolescente permitió la identificación de categorías de acuerdo con las variables del modelo. En el proceso de ajuste del adolescente se reconocieron dimensiones de ajuste psicosocial y síntomas de respuesta al estrés, como rendimiento académico y síntomas somáticos. Las intervenciones de enfermería se centrarán en la educación y el apoyo.

**Conclusión:**

El modelo teórico contribuyó para evaluar las necesidades de los adolescentes que experimentan cáncer en los padres, permitiendo que las intervenciones de enfermería sean prescritas en un lenguaje clasificado que considere variables moderadoras y mediadoras, promoviendo el ajuste. El modelo demostró ser adecuado para futuras intervenciones con adolescentes en situaciones similares.

## INTRODUCTION

The incidence rate of cancer in young adults has increased significantly in recent decades. Internationally, it is estimated that 15% of people with cancer are between 20 and 50 years old^(1)^.

Parental cancer comprises the experience of cancer by patient and family, being a stressful and disturbing experience for the whole family, given the nature of disease, its physical and psychological consequences, uncertainty about the future and potential for threat of death, which can cause suffering and changes in the binomial interaction^(2)^. It can be mainly challenging for children and adolescents^(3)^.

Adolescents are a particularly vulnerable population when faced with parental cancer, showing anxiety and depression^(2,4)^. The development of abstract thinking and increased cognitive skills make them more susceptible to distress, as they are more aware of consequences of cancer, such as loss, physical and emotional pain for parents^(2)^. Adolescents living with a parent with cancer have a higher risk of internalization (depression, anxiety, somatic symptoms), externalization problems (aggressive behaviors, delinquents), lower quality of life and changes in school performance^(4)^.

Considering the repercussions and challenges that the disease poses, not only to the sick but also to the children, an inclusive nursing intervention by the binomial^(5)^ is necessary. However, despite the impact of cancer on the parent-child binomial, there are no specific guidelines regarding care and relationship with families experiencing such a reality^(6)^.

As a conceptual basis for the nursing intervention, A Model of Children’s Adjustment to Parental Cancer (AMCAPC) was reference standard, proposed by Su and Ryan-Wenger^(7)^. Although the evidence identifies at least two models, only AMCAPC incorporates the nursing intervention. AMCAPC shows how important concepts, such as adjustment and adaptation, are related and how some variables moderate or mediate adjustment to the experience of parental cancer^(7)^. Moreover, it presents as philosophical principles Piaget’s theory of cognitive development, Lazarus & Folkman’s Stress and Coping theory and Bowen’s Family Systems theory^(7)^.

This study aimed to describe and analyze the experience of parental cancer by an adolescent, from AMCAPC use, and prescribe nursing interventions using classified language.

## METHOD

### Study Design

This is a single case study, which is part of the qualitative type paradigm, related to adolescents’ adjustment to the experience of parental cancer. To develop this study, we used the COREQ checklist.

### A Model of Children’s Adjustment to Parental Cancer

**Figure 1.**
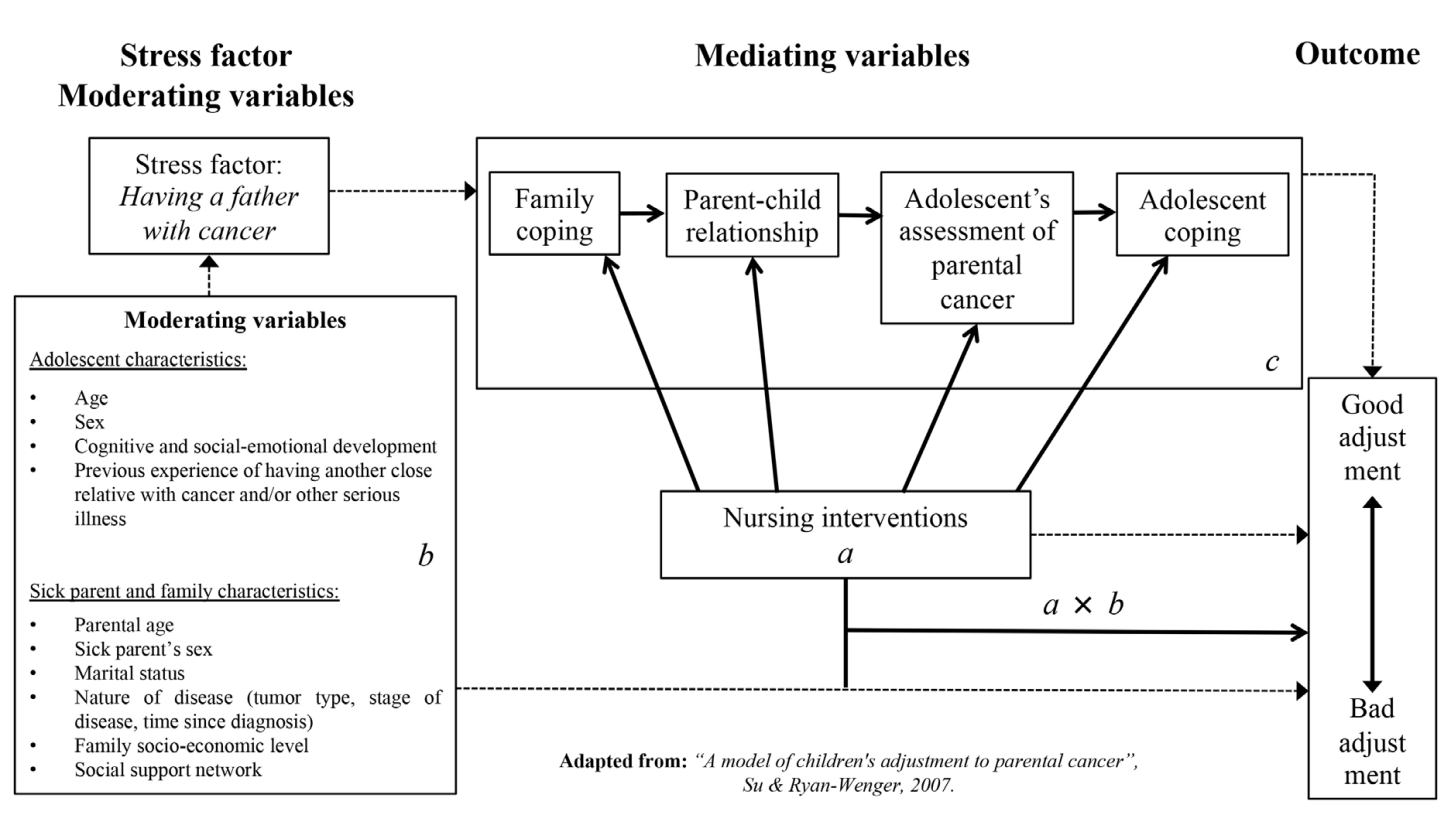
A Model of Children’s Adjustment to Parental Cancer^(7)^.

AMCAPC states that the diagnosis of cancer invariably leads to psychological and social stress in adolescents and that the factors that contribute to adolescents’ adaptation can be classified as moderating and mediating factors. The preexisting variables that influence the situation that generates stress were called moderating, and the variables that exert their influence after the diagnosis of the parental disease, mediating^(7)^. Moderating variables in adolescents include characteristics such as age, sex, cognitive and socio-emotional development, and previous experiences of another family member with cancer and/or other serious illness. Moderating variables in the parent include age, sex, marital status and nature of disease (tumor type, disease stage, time since diagnosis), family socioeconomic status, and social support network (family/other)^(7)^. Mediating variables integrate four fundamental concepts: family coping, parent-child relationship, adolescent assessment of parental cancer and adolescent coping strategies^(7)^. The nursing interventions recommended by the model meet the mediating variables. These are influenced by the intervention and the outcome is the response to the interaction of moderating and mediating variables and nursing interventions. Consequently, there may be a good or bad adolescents’ adjustment^(7)^. Nursing interventions comprise three components: education (on parental cancer), normalization (creating a safe environment that allows the expression of feelings, providing psychological support) and strength development (helping to recognize the ability to deal with stress events that allow developing coping mechanisms)^(7)^. Figure 1 illustrates AMCAPC, demonstrating the interaction between stress factor, moderating and mediating variables and outcome.

### Place

The study was conducted in an oncology hospital in central Portugal.

### Period

Data collection was carried out on February 26, 2021.

### Choice of Sample

Adolescent male, 16 years old, attending the 11^th^ grade of secondary education.

### Selection Criteria

Being an adolescent, between 14 and 18 years old, son of a cancer patient without cognitive deficits, who understands and speaks Portuguese fluently. The adolescent and his parents signed the Informed Consent Form (ICF) to participate in the study. Adolescent selection occurred due to ease of access through the mother with cancer.

### Instruments used for Data Collection

Data collection carried out using a semi-structured interview script^(8)^, based on the methodological principles and concepts presented in AMCAPC^(7)^.

### Data Collection

Data collection was carried out in a virtual environment, through Colibri-Zoom, due to the pandemic period, with a view to maintaining the interviewees’ safety. The interview took place in a calm environment, selected by the adolescent, his bedroom. After the researcher was introduced to the adolescent, in the presence of his mother, the interview was conducted only between the participant and the interviewer, and lasted 30 minutes. The video audio was recorded, and after its transcription, destroyed.

### Data Treatment and Analysis

Data were coded and categorized by units of meaning, according to the content analysis framework^(9)^, based on the AMCAPC theoretical framework^(7)^. Categorization occurred inductively, to identify dimensions or themes, through the interview analysis^(9)^. The software MAXQDA was used.

### Ethical Aspects

This research was approved by the Research Ethics Committee of the institution where the study was developed (Process TI 25/2020). The adolescent and his legal guardian (mother) consented to the participation and recording of the interview, signing the ICF. Participants’ statements were coded in order to safeguard data anonymity and confidentiality, identifying themselves as A1.

## RESULTS

The participant is a 16-year-old male adolescent. In agreement with Piaget, the mother is in the formal operations stage, showing the ability to reflect on her current situation, daily life activities, formulate hypotheses, demonstrate autonomy over choices and decisions and understand the situation she is experiencing. According to AMCAPC, the stressor is having a mother with cancer. Moderator variables include adolescent characteristics and sick parent and family characteristics. The household consists of four members, the mother, 50 years old, married to the adolescent’s father, 53 years old, and the sister, 20 years old. The adolescent has previous experience of family with cancer, his paternal grandmother. The family socioeconomic level has not changed in status, and the family is the social support network. Regarding the nature of disease, his mother has invasive breast carcinoma, stage 2, diagnosed one year ago. At the time of the interview, she was undergoing neoadjuvant treatments (chemotherapy, hormone therapy and immunotherapy) and postoperative recovery (mastectomy).

**Chart 1:**
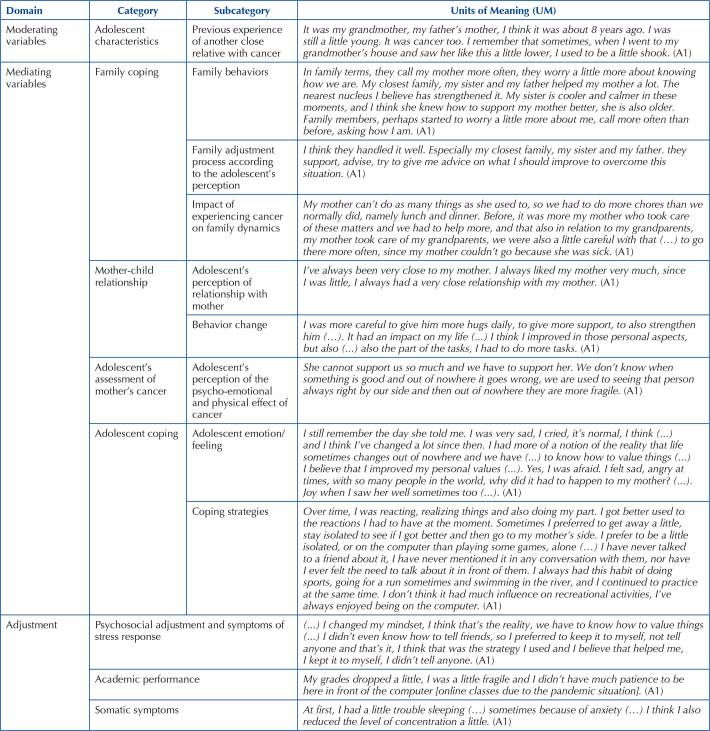
Domains, categories, subcategories and units of meaning – Coimbra, Portugal, 2021.

As for mediating variables, we identified contents of the adolescent’s communication that fit into all the categories conceptualized in the model and whose outcomes are presented in Chart 1. In mediating variable “adolescent’s assessment of mother’s cancer”, according to the analysis of the adolescent’s interview and according to the model, the experience of parental cancer was classified as “stressful”, with a “challenge” response, since, although the experience of parental cancer causes stress, there was an opportunity for the adolescent to grow and gain. In mediating variable “adolescent coping”, it is identified that it presented coping focused on the problem and emotion, seeking to adjust to the situation and normalize the emotions related to the mother’s cancer (stress factor). Based on the adolescent’s interview and analysis of moderating and mediating variables, we identified the dimensions of psychosocial adjustment and stress response symptoms, academic performance and somatic symptoms.

**Caption:** (…) – Excerpts from the original transcript irrelevant to our analysis.

**Figure 2.**
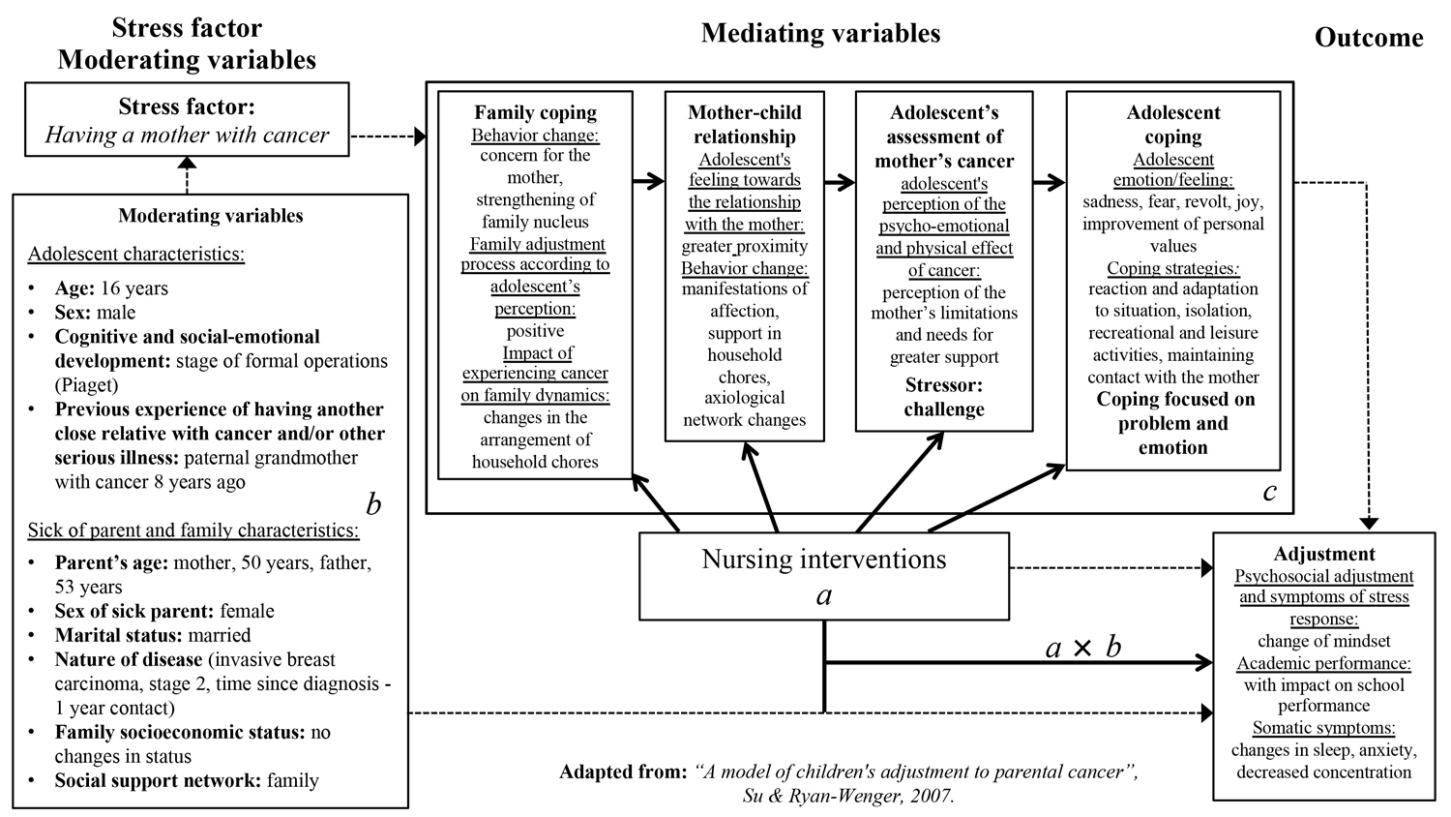
A Model of Children’s Adjustment to Parental Cancer, adapted by the authors to the case under study (original diagram: Su & Ryan-Wenger^(7)^).

Figure 2, based on AMCAPC and adapted to the case under study, it schematically represents the interaction of moderating and mediating variables and the prescribed nursing interventions, which may result in a good or bad adjustment.

According to AMCAPC, nurses, in planning nursing care, identify the nature of the stress factor and the interaction of moderating and mediating factors, assessing its real and potential effects, in order to, in partnership with the adolescent and family, establish a care plan adapted to their needs. By assessing the adolescent’s needs, “knowledge”, “sadness”, “fear”, “anxiety”, “adaptation” and “family coping” are the nursing focuses in need of more urgent intervention in care planning.

**Chart 2:**
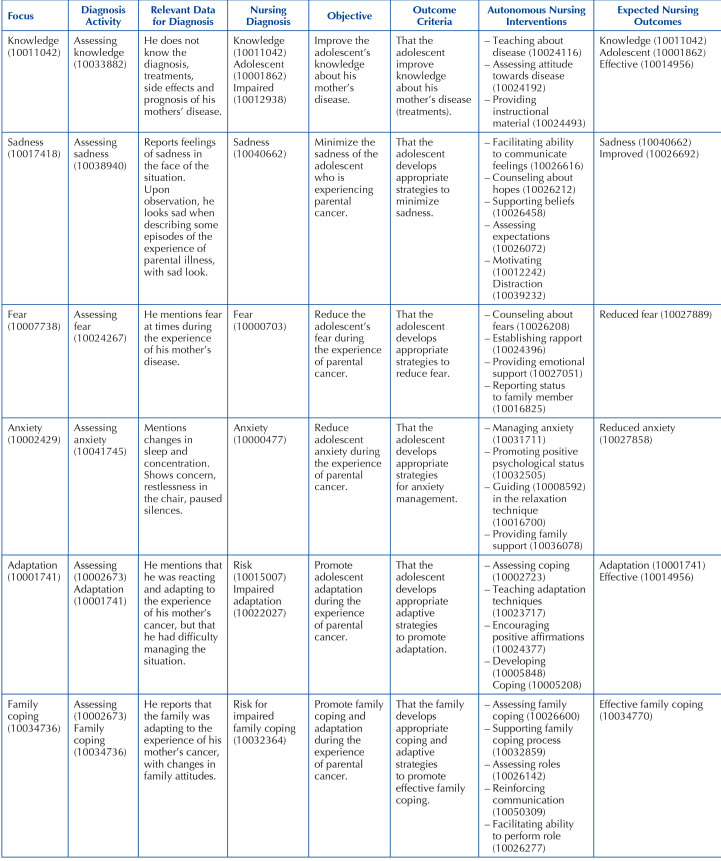
Care plan based on ICNP^®(10)^ taxonomy – Coimbra, Portugal, 2021.

Thus, a nursing care plan was elaborated using the ICNP^®^^(10)^ taxonomy, with the presentation of the respective coding for the focuses, nursing diagnoses (ND), interventions, diagnostic and assessment activities, and expected nursing outcomes, according to Chart 2. The use of this classification supports the decision-making process, facilitating systematic documentation and communication in the nursing team^(11)^.

## DISCUSSION

The analysis of the interview allowed to know the adolescent’s experience, organizing the outcomes in the categories of the mediating variables: *Family coping*; *Mother-child relationship*; *Adolescent’s assessment of mother’s cancer*; *Adolescent coping*; *Nursing interventions*; and *Adjustment*.

### Family Coping

The adolescent’s perception of family adjustment process was positive, stating that they knew how to adapt and face the situation. However, the experience of parental cancer caused changes, showing changes in family behavior, demonstrating greater concern and strengthening of family support networks. When a family is faced with parental cancer, there can be changes and difficulties that impair the quality of life of parents and children, causing consequences in different dimensions^(4)^. It was found that the experience of cancer affected the dynamics of this family, with changes in the adolescent’s, his sister’s and his father’s behavior, in terms of responsibility for household chores and caring for maternal grandparents, with the three elements taking on roles that were once taken on by his mother. These changes can affect the quality of life of the entire family unit in the emotional, social, physical, spiritual and financial dimensions^(12)^. According to what was mentioned by the adolescent, the dimensions most affected were emotional, social and physical, due to the mother’s wear and tear, given the consequences of neoadjuvant treatments, determining changes in roles in the entire family structure.

### Mother-Child Relationship

The adolescent reported having a close and affectionate relationship with his mother, showing a change in behavior after the diagnosis, with greater emotional support to the mother and collaboration in chores.

The diagnosis and development of cancer in one of the parents cause changes in family functioning, causing behavioral, emotional and physical changes in the adolescent^(4)^. One of the changes mentioned by the adolescent was to take responsibility for chores that he did not previously perform. Scientific evidence points to role reversal as a consequence of parental cancer in adolescents’ family life patterns^(4)^. The adolescent, despite mentioning that performing household chores did not interfere with school activities or relationship with the peer group, considered that his school performance was affected; however, he related this fact to changes in the emotional dimension. The quality of communication between parents and children, associated with a “warm parenting”, can play a protective role in adolescents’ adjustment to parental cancer, promoting more effective coping and strengthening existing relationships^(13)^. In the case under study, Despite the changes in family dynamics caused by parental cancer, the positive relationship of closeness with the mother before the diagnosis, the existence of open communication and the ability to maintain activities allowed to preserve and strengthen the binomial’s relationship.

### Adolescent’s Assessment of Mother’s Cancer

The adolescent identified his mother’s limitations, resulting from the psycho-emotional and physical effects of cancer, implying a greater need for support. The situation was classified as “stressful”, with a response of “challenge”, because, although the experience of cancer generates stress, there was an opportunity for growth, providing the adolescent with a greater sense of reality, valuing life and improving personal values. The mother’s cancer assessment is influenced by the transition process inherent in the adolescent’s growth/development, corroborating other authors, who state that adolescents who experience these situations live under constant pressure, struggle for independence and face the need to support parents, both physically and emotionally^(12)^. The adolescent under study did not report changes in the relationship with peers, but did not share any of his experience, referring to lack of knowledge about how to report. This position can be justified, considering that some adolescents may find it difficult to communicate and express their emotions, not sharing their experiences out of fear or to suppress their own feelings^(14)^.

### Adolescent Coping

The adolescent under study demonstrated coping focused on the problem, reacting and adapting to the situation, since it changed some of its behaviors, taking on greater responsibility for chores. Simultaneously, he presented emotion-focused coping, seeking to normalize emotions related to the mother’s cancer through isolation, which he resorted to in some periods, keeping in touch with the mother, choosing not to share the situation with the peer group and refusing to talk and think about the problem. Some authors^(14)^ report that adolescents have different coping mechanisms in the experience of parental cancer, such as talking about the situation and coping with it, or blocking the topic, avoiding thinking more about it, being able to show different strategies, such as spending more time with their parents and, sometimes, isolating themselves in their space. Both strategies were adopted at different times by the adolescent under study, reporting distraction as a coping mechanism, resorting to playful activities with friends, physical activities, or doing something fun.

### Adjustment

The adolescent under study is in late adolescence (16-19 years)^(15)^, considered a critical period that is characterized by changes in individuals’ life context. In this age group, it is normal to be concerned about the impact of parental illness on their daily activities, especially school, sports/leisure activities, and may present changes in school performance, physical complaints of pain, discomfort and social and interpersonal changes^(4)^. In the case under analysis, we evidenced some of these alterations, particularly in terms of psychosocial adjustment dimensions and stress response symptoms, such as changes in thinking, academic performance, impact on school performance and somatic symptoms, with changes in sleep, anxiety and decreased concentration.

One way to minimize the psychosocial consequences of experiencing parental cancer and enhance adjustment is to meet their needs for information about their parents’ cancer; family functioning; professional assistance; “time out” and playful activities; dealing with feelings; support from the peer group and other young people who have gone through similar situations^(4)^. In this case, it appears that some needs have been met, namely information on the mother’s cancer, through open communication, enhanced by the positive relationship between family members, specifically between mother and child. It was found that the adolescent was able to maintain playful activities and connection with peer group, referring to personal growth in learning to deal with their feelings.

### Nursing Interventions

According to AMCAPC, nursing interventions incorporate essential elements at the level of education, normalization and development of strengths, which are in line with the adolescent’s needs. Despite the adolescent having an open communication with his mother, he was not able to share with peer group what he was experiencing, evidencing the need for information, which is why he identified himself in the “knowledge” focus. The need for information and knowledge about the parents’ cancer diagnosis, treatment implications and prognosis is mentioned by other authors as the psychosocial need most strongly reported by adolescents^(2,4)^. For the “sadness” and “fear” NDs, we prescribe interventions that contribute to normalization, through the creation of a safe environment that allows the expression of emotions that, enhanced by the maximization of effective communication, promotes adjustment^(14)^. The prescribed nursing interventions related to “anxiety” ND aim to enable the adolescent to develop their strengths, supporting them to recognize and deal with stressful events. The “adaptation” and “family coping” will allow the adolescent, together with his family, to adjust to parental cancer. A study^(16)^ on intervention programs for children, adolescents and parents experiencing parental cancer identified that the dominant interventions are psychoeducational. The typology of these interventions integrates the psychological/emotional and educational component, aiming to provide social support to families, increase parenting skills, improving children’s understanding of cancer, reducing anguish and fears^(16)^. These interventions are considered appropriate, contributing to increase health literacy, promote the expression of emotions/experiences, assist in changing roles, improve anxiety symptoms, develop coping mechanisms and optimize communication and parent-child relationship.

Considering that adjustment is based on nursing interventions, it is expected that the adolescent presents a “good adjustment”, because, although consequences are verified, he demonstrated the ability to adjust to the situation, seeing it as a challenge that resulted in an opportunity for growth.

### Study Limitations

The single-case research has methodological potential in minimizing bias, but the analysis of a single case may constitute a limitation in the generalization of the findings to other adolescents.

### Implications for Practice

The results of this study may be applied in similar situations of adolescents who experience parental cancer. Nurses who integrate interventions that respond to the needs of adolescents and families experiencing parental cancer, promoting adjustment, contribute to minimizing the psychosocial impact resulting from this situation. The model is applicable and suitable for adolescents going through similar situations, allowing the identification of nursing focuses and the consequent prescriptions and interventions that respond to the priority needs of adolescents with parents with cancer.

## CONCLUSION

AMCAPC has been shown to be relevant in assessing the needs of an adolescent experiencing parental cancer. The analysis of this case made it possible to identify parental cancer as a stress factor, through the interaction of moderating and mediating variables, which, together with the prescribed nursing interventions, can promote the “good adjustment” of adolescents experiencing parental cancer. We suggest carrying out studies in contexts of experiencing parental cancer, using AMCAPC, in which nursing interventions are implemented and assessed.
